# Efficacy of Hormonal and Nonhormonal Approaches to Vaginal Atrophy and Sexual Dysfunctions in Postmenopausal Women: A Systematic Review

**DOI:** 10.1055/s-0042-1756148

**Published:** 2022-11-29

**Authors:** Ayane Cristine Alves Sarmento, Ana Paula Ferreira Costa, Juliana Lírio, José Eleutério Jr, Pedro Vieira Baptista, Ana Katherine Gonçalves

**Affiliations:** 1 Postgraduate Program student in Health Science, Universidade Federal do Rio Grande do Norte, Natal, RN, Brazil; 2 Department of Obstetrics and Gynecology, Universidade Estadual de Campinas, Campinas, SP, Brazil; 3 Department of Obstetrics and Gynecology, Universidade Federal do Ceará, Fortaleza, CE, Brazil; 4 Lower Genital Tract Unit, Centro Hospital de São João, Porto, Portugal

**Keywords:** menopause, dyspareunia, orgasm, therapeutics, estrogens, menopausa, dispareunia, orgasmo, terapêutica, estrogênios

## Abstract

**Objective** To evaluate the efficacy of the hormonal and nonhormonal approaches to symptoms of sexual dysfunction and vaginal atrophy in postmenopausal women.

**Data Sources** We conducted a search on the PubMed, Embase, Scopus, Web of Science, SciELO, the Cochrane Central Register of Controlled Trials (CENTRAL), and Cumulative Index to Nursing and Allied Health Literature (CINAHL) databases, as well as on clinical trial databases. We analyzed studies published between 1996 and May 30, 2020. No language restrictions were applied.

**Selection of Studies** We selected randomized clinical trials that evaluated the treatment of sexual dysfunction in postmenopausal women.

**Data Collection** Three authors (ACAS, APFC, and JL) reviewed each article based on its title and abstract. Relevant data were subsequently taken from the full-text article. Any discrepancies during the review were resolved by consensus between all the listed authors.

**Data Synthesis** A total of 55 studies were included in the systematic review. The approaches tested to treat sexual dysfunction were as follows: lubricants and moisturizers (18 studies); phytoestrogens (14 studies); dehydroepiandrosterone (DHEA; 8 studies); ospemifene (5 studies); vaginal testosterone (4 studies); pelvic ﬂoor muscle exercises (2 studies); oxytocin (2 studies); vaginal CO _2_ laser (2 studies); lidocaine (1 study); and vitamin E vaginal suppository (1 study).

**Conclusion** We identified literature that lacks coherence in terms of the proposed treatments and selected outcome measures. Despite the great diversity in treatment modalities and outcome measures, the present systematic review can shed light on potential targets for the treatment, which is deemed necessary for sexual dysfunction, assuming that most randomized trials were evaluated with a low risk of bias according to the Cochrane Collaboration risk of bias tool. The present review is registered with the International Prospective Register of Systematic Reviews (PROSPERO; CRD42018100488).

## Resumo

**Objetivo** Avaliar a eficácia das abordagens hormonais e não hormonais para os sintomas de disfunção sexual e atrofia vaginal em mulheres na pós-menopausa.

**Fontes de Dados** Pesquisamos as bases de dados PubMed, Embase, Scopus, Web of Science, SciELO, Cochrane Central Register of Controlled Trials (CENTRAL), e Cumulative Index to Nursing and Allied Health Literature (CINAHL), assim como bancos de dados de ensaios clínicos. Foram analisados estudos publicados entre 1996 e 30 de maio de 2020. Nenhuma restrição de idioma foi aplicada.

**Seleção dos Estudos** Foram selecionados ensaios clínicos randomizados que avaliavam o tratamento das disfunções sexuais em mulheres na pós-menopausa.

**Coleta de Dados** Três autores (ACAS, APFC e JL), revisaram cada artigo com base em seu título e resumo. Os dados relevantes foram posteriormente retirados do texto completo do artigo. Quaisquer discrepâncias durante a revisão foram resolvidas por consenso entre todos os autores listados.

**Síntese dos Dados** Ao todo, 55 estudos foram incluídos na revisão sistemática. As abordagens testadas para tratar a disfunção sexual foram: lubrificantes e hidratantes (18 estudos); fitoestrogênios (14 estudos); deidroepiandrosterona (DHEA; 8 estudos); ospemifeno (5 estudos); testosterona vaginal (4 estudos); exercícios para os músculos do assoalho pélvico (2 estudos); oxitocina (2 estudos); *laser* de CO _2_ vaginal (2 estudos); lidocaína (1 estudo), e vitamina E vaginal (1 estudo).

**Conclusão** Identificou-se falta de coerência na literatura quanto aos tratamentos propostos e medidas de resultados selecionadas. Apesar da grande diversidade de modalidades de tratamento e medidas de resultados, esta revisão sistemática pode lançar luz sobre alvos potenciais para o tratamento, que é considerado necessário para a disfunção sexual, assumindo que a maioria dos estudos randomizados foi avaliada com baixo risco de viés de acordo com a ferramenta de avaliação de risco de viés de Cochrane Collaboration. Esta revisão tem cadastro no International Prospective Register of Systematic Reviews (PROSPERO; CRD42018100488).

## Introduction

According to the North American Menopause Society (NAMS), natural menopause is defined as the final menstrual period, diagnosed after 12 consecutive months of spontaneous amenorrhea without an apparent pathological cause. The most common symptoms associated with menopause are hot flushes, night sweats, sleep disturbance, vaginal atrophy, and dyspareunia. 1 2

Studies 3 4 5 6 7 8 indicate that postmenopausal women with vulvovaginal atrophy (VVA) have a higher probability of developing sexual dysfunction, including difficulties regarding sexual desire, arousal, lubrication, and orgasm. Vulvovaginal atrophy leads to thinning of the mucus and tissues of the vulva and vagina caused by the estrogen deprivation that occurs in women during this period. Patients with VVA complain of vaginal irritation and discharge, itching, dryness, dysuria, and dyspareunia. Despite the decrease in sexual function indices during the menopausal transition, 3 it is not clear whether this is caused by lower levels of ovarian hormones, aging, or both. 4

According to the American Psychiatric Association (APA), 5 6 7 8 sexual dysfunction is defined as disorders of sexual desire and psychophysiological changes that characterize the sexual-response cycle, causing marked suffering and interpersonal difficulty. Female sexual dysfunction (FSD) can be assessed in different domains, including sexual interest, arousal, orgasm, and pain,9 and is not easy to define or investigate, because it depends on several factors, such as health and well-being, cultural habits, socioeconomic status, relationship problems, and sexual partner's existence or not. 5

The measurement of sexual function in women has never been an easy task. Furthermore, the literature 6 describes a diverse assortment of outcome measures across various symptom levels, and, as a result, pooling of data is imprecise.

Because of the complexity of FSD, it is often difficult to define the factors primarily responsible for the disorder and to establish meaningful steps in the treatment. The treatments usually aim to deal with individual symptoms, but no single treatment modality addresses the entire spectrum of the disorder. Therefore, multimodal therapy is required. The great variety of distinct classes of medication described in the literature 7 confirms the complexity of the disorder and indicates the need for a systematic review to define the best practices.

Several options are available to treat this condition, and hormonal therapies (HTs; estrogens and androgens) and nonhormonal therapies (non-HTs; lubricants and long-acting vaginal moisturizers) are the most commonly used. It is recognized that vaginal estrogen may improve the symptoms; conversely, non-HTs can be useful in specific cases in which HTs are harmful or not recommended (such as breast cancer). 7 10

The real effect of the treatments on sexual function in menopausal women is particularly difficult to understand based on the literature published so far, partly because of different drugs, routes, and dosages, as well as the diversity of tools used to assess sexual function and the studied population in each trial. Similarly, it is essential to have guidance on effective alternative treatments for women who cannot use HTs. Thus, there is a real need for a systematic review of the best available evidence to facilitate evidence-based decisions. Thus, the present systematic review aimed to evaluate the efficacy of hormonal and nonhormonal approaches for symptoms of sexual dysfunction and vaginal atrophy in postmenopausal women.

## Methods

### Study Design, Data Search, and Inclusion and Exclusion Criteria

The present systematic review followed the Preferred Reporting Items for Systematic Reviews and Meta-Analyses (PRISMA) statement 11 and has been registered with PROSPERO (CRD42018100488). Ethical approval was not required because the review uses published patient data. A full systematic search was carried out on the following databases: PubMed, Embase, Scopus, Web of Science, SciELO, the Cochrane Central Register of Controlled Trials (CENTRAL), and Index to Nursing and Allied Health Literature CINAHL, as well as clinical trial databases ( www.trialscentral.org ; www.controlled-trials.com ). We included studies published from 1996 until May 30, 2020 without language restrictions. Grey literature was not searched. Combinations of the following keywords were used to identify the studies: *menopause* ; *postmenopause* ; *dyspareunia* ; *orgasm* ; *therapeutics* ; *dehydroepiandrosterone* ; *testosterone* ; *oxytocin* ; *phytoestrogens* ; *lidocaine* ; *hyaluronic acid* ; *lubricants* ; and *laser* . The PubMed search strategy is shown in Chart 1 .

**Chart 1:** Search strategy on PubMed

Number	Search items
**1**	Menopause
**2**	Postmenopause
**3**	Genitourinary syndrome of menopause
**4**	Dyspareunia
**5**	Orgasm
**6**	Vaginal atrophy
**7**	Female sexual dysfunction
**8**	Sexual function
**9**	Or/1-8
**10**	Therapeutics
**11**	Vaginal estrogen
**12**	Estrogens
**13**	Vaginal therapy
**14**	Non-hormonal treatments
**15**	Dehydroepiandrosterone
**16**	Testosterone
**17**	Oxytocin
**18**	Phytoestrogens
**19**	Local lidocaine
**20**	Hyaluronic acid
**21**	Lubricants
**22**	Moisturizers
**23**	Laser therapy
**24**	Radio waves
**25**	Microablative fractional radiofrequency
**26**	Physical therapy modalities
**27**	Or/10-26
**28**	Randomized clinical trial
**29**	Clinical trial
**30**	Or/28-29
**31**	9 and 27 and 30

Three authors (ACAS, APFC, and JL) reviewed each article based on its title and abstract. The relevant data were subsequently taken from the full-text article. We evaluated the full text based on the following inclusion criteria: 1) studies with postmenopausal women; 2) randomized clinical trials evaluating the efficacy of hormonal or nonhormonal approaches to sexual dysfunction; 3) intervention groups undergoing treatment for sexual dysfunction; 4) any HT (considering HTs, according to the NAMS, as all the prescription drugs used most often to treat hot flashes and symptoms of genitourinary syndrome of menopause [GSM], which include vaginal dryness) or non-HT being compared to a placebo or no intervention or vaginal estrogen (all types of estrogen, progesterone, and androgens); 1 and 5) improvement in sexual dysfunction as the primary outcome. Cross-sectional and observational studies were excluded, and published studies were excluded if only their abstract was available.

The full texts of potentially-eligible studies were extracted and examined for the following data: year the study was conducted; number of subjects; location of subject recruitment; mean age of the subjects; randomization and blinding processes; inclusion and exclusion criteria; description of the therapies used, and definition of the outcomes measured; length of follow-up; and side effects. Any discrepancies during the review were resolved by consensus between all the listed authors.

### Primary and Secondary Outcomes

The primary outcomes were vaginal dryness, arousal, desire, orgasm, lubrication, satisfaction, pain, and dyspareunia. The secondary outcomes included side effects, treatment duration, and adhesion. The outcomes of the randomized controlled trials included had to be measured using gynecological and visual examinations, pH, cell maturation index, and validated scales. For the secondary outcomes, the side effects were evaluated according to the records of their onset and throughout treatment by self-assessment questionnaires.

### Assessment of the Risk of Bias and Qualitative Analysis

To assess the risk of bias, the Cochrane Collaboration risk of bias tool was applied to evaluate the following criteria: adequate sequence generation; allocation concealment; blinding; incomplete outcome data; selective reporting; and other risks of bias. 12 Three authors assessed each original study, and the quality of the data is shown in Chart 2 . Relevant data were subsequently extracted from the full-text article, according to the data extraction protocol. Each of the aforementioned criteria received one of the following classifications: “low risk of bias”; “high risk of bias”; or “unclear risk of bias”. Disagreements were resolved by consulting a third author. Moreover, regarding the primary outcomes, we assessed the certainty of the evidence according to the Grading of Recommendations Assessment, Development, and Evaluation (GRADE) classification. 13

**Chart 2:** Quality assessment of the included studies using the Cochrane risk of bias tool

Study	Random sequence generation	Allocation concealment	Blinding of participants and personnel	Blinding of outcome assessment	Incomplete outcome data	Selective reporting	Other bias
Bygdeman and Swahn (1996) 14							
Loprinzi et al. (1997) 15							
Balk et al. (2002) 32							
Labrie et al. (2009) 45							
Bachmann and Komi (2010) 52							
Oh et al. (2010) 33							
Raghunandan et al. (2010) 16							
Ekin et al. (2011) 67							
Genazzani et al. (2011) 46							
Jonasson et al. (2011) 61							
Labrie et al. (2011) 27							
Le Donne et al. (2011) 28							
Lee et al. (2011) 17							
Loprinzi et al. (2011) 18							
Amato et al. (2013) 34							
Grimaldi et al. (2012) 29							
Tedeschi and Benvenuti (2012) 35							
Chen et al. (2013) 68							
Lima et al. (2013) 36							
Portman et al. (2013) 53							
Zheng et al. (2013) 30							
Constantine et al. (2015) 54							
Fernandes et al. (2014) 19							
Labrie et al. (2014) 47							
Lima et al. (2014) 37							
Portman et al. (2014) 55							
Archer et al. (2015) 48							
Bouchard et al. (2015) 49							
Goetsch et al. (2015) 65							
Labrie et al. (2015) 50							
Tungmunsakulchai et al. (2015) 57							
Hickey et al. (2016) 20							
Jokar et al. (2016) 31							
Juliato et al. (2017) 21							
Labrie et al. (2016) 51							
Melisko et al. (2017) 58							
Postigo et al. (2016) 38							
Seyyedi et al. (2016) 22							
De Souza et al. (2016) 39							
Yaralizadeh et al. (2016) 40							
Cruz et al. (2018) 63							
Malakouti et al. (2017) 41							
Nappi et al. (2017) 23							
Nazarpour et al. (2017) 59							
Suwanvesh et al. (2017) 42							
Diem et al. (2018) ^24^							
Golmakani et al. (2019) 66							
Mitchell et al. (2018) 25							
Nazarpour et al. (2018) 60							
Torky et al. (2018) 62							
Archer et al. (2019) 56							
Ghorbani et al. (2019) 43							
Mitchell et al. (2019) 26							
Palma et al. (2019) 44							
Politano et al. (2019) 64							

Key:  High risk of bias;  Unclear risk of bias;  Low risk of bias

As for the quantitative studies, we could not perform a formal statistical analysis because of the heterogeneity of the measurements. However, the findings relevant to the aims of the present review were extracted.

## Results

### Study Selection and Characteristics

**Fig. 1:**
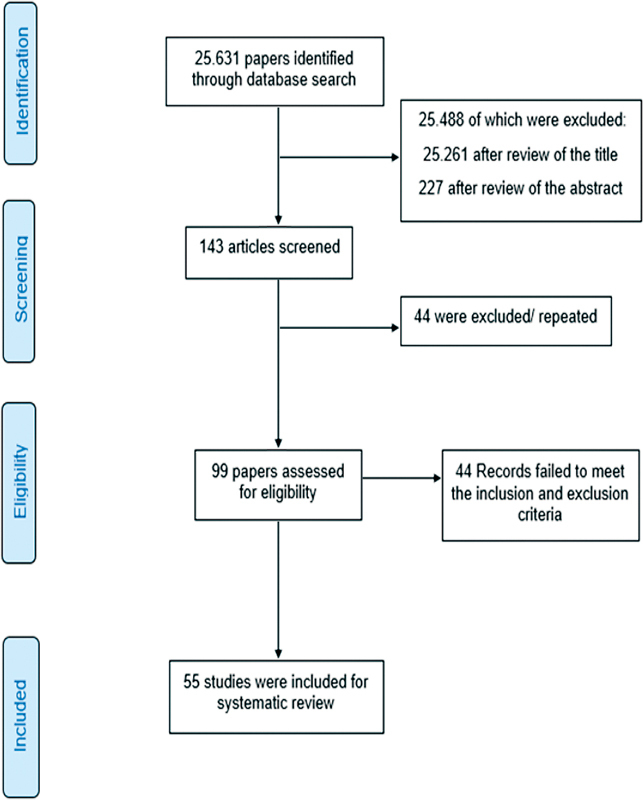
Preferred Reporting Items for Systematic reviews and Meta-Analyses (PRISMA) diagram of the search strategy used in the present systematic review.

After searching the databases, 25,631 articles were identified ( Figure 1 ). After a review of their titles and abstracts, 25,488 records were excluded, and 143 records remained for full-text review. In total, 44 articles were excluded because they were duplicates. After reviewing the full-text articles, an additional 44 records were excluded. This process resulted in 55 articles, which were further reviewed using a manual search approach.

A total of 55 studies were deemed eligible for inclusion ( Table 1, Supplementary material ). The approaches tested to treat vaginal atrophy and sexual dysfunction were as follows: lubricants and moisturizers (18 studies, 14 15 16 17 18 19 20 21 22 23 24 25 26 5 27 28 29 30 31 of which evaluated hyaluronic acid); phytoestrogens (14 studies); 30 32 33 34 35 36 37 38 39 40 41 42 43 44 dehydroepiandrosterone (DHEA; 8 studies); 27 45 46 47 48 49 50 51 ospemifene (5 studies); 52 53 54 55 56 vaginal testosterone (4 studies); 16 19 57 58 pelvic ﬂoor muscle exercises (2 studies); 59 60 oxytocin (2 studies); 61 62 vaginal CO _2_ laser (2 studies); 63 64 lidocaine (1 study); 65 and vitamin E vaginal suppository (1 study). 66

Overall, 18 studies evaluated the parameters of dyspareunia and vaginal dryness, 2 evaluated symptoms of VVA, and the other studies used validated questionnaires for the evaluation of sexual dysfunction.

### Side Effects

Regarding side effects, the results for lubricants and moisturizers indicated that all adverse events (AEs) were considered mild in severity and self-limited. 14 15 16 17 18 19 20 21 22 23 24 25 26 The most common AEs were burn on the application site, discomfort on the application site, vulvovaginal pruritus, and general pruritus. The compound hyaluronic acid has been shown to be well tolerated without side effects among most patients tested. 28 29 31 67 68

For *soy* flour and *fennel* cream, no adverse effects were reported in any of the included studies. 32 40 *Cimicifuga foetida* extract was found to be effective and safe during three months of treatment. 30 For *ginseng* , 12 events were reported during a clinical trial; 33 of these, 2 were of vaginal bleeding, which were assumed to be possibly related to the trial. Among the studies selected, there were only two reports of participants experiencing serious AEs. 34 35 36 37 For *Tribulus terrestris* , the most frequent adverse effects were diarrhea, nervousness, dizziness, and nausea. 38 39 In relation to the other phytoestrogens, the studies did not provide data on side effects.

Approximately half of the subjects treated with DHEA experienced the most frequently reported treatment-emergent AEs (TEAEs). The three most commonly reported preferred terms were *discharge at the application site* , *urinary tract infection* , and *headache* . No adverse effects were observed regarding hepatic tests, hematocrit, or any hematological or biochemical parameters. 27 45 46 47 48 49 50 51

Ospemifene was shown to be safe and well tolerated. Most TEAEs were classified as not related to the study drug or were unlikely to be related. The most frequent were urinary tract infection, hot flush, and nasopharyngitis. Only two patients experienced serious AEs, one of which was probably treatment-related (deep vein thrombosis). 52 53 54 55 56

All related AE treatments with vaginal testosterone were classified grade 1 (mild) or 2 (moderate). The most frequent were vaginal discharge, facial hair growth, vaginal or vulvar itching and/or irritation, vaginal odor, and urinary tract or yeast infection. 16 19 57 58

None of the study participants reported any side effects related to the use of oxytocin. 61 62 There were no data available on the side effects of the treatment with pelvic ﬂoor muscle exercises, 59 60 vaginal CO _2_ laser, 63 lidocaine, 65 and vitamin E vaginal suppository. 66

## Discussion

The female population is aging and facing new health issues, including menopause symptoms and FSD. Despite this, the best approach to sexual dysfunction in menopausal women is still unclear.

Until now, estrogens and androgens for FSD have been shown to improve not only dyspareunia and vaginal dryness but also the desire and orgasm domains. However, not all women can use them; breast cancer, endometrial cancer, and deep venous thrombosis, among other conditions, can be contraindications for HTs. Moreover, there is insufficient evidence to attest to the effectiveness of the non-HTs studied to improve orgasm, lubrication, and sexual satisfaction in menopausal women. 1 2

The present review suggests that the use of lubricants and moisturizers has a lower impact than that of vaginal estrogens. The domains in which improvement was more evident were dyspareunia and vaginal dryness. The results of the present review point out the efficacy of hyaluronic acid for the symptoms of vaginal atrophy when compared with vaginal estrogen. Previous studies by Ekin et al. 67 and Chen et al. 68 have also demonstrated its effectiveness. Laser therapy, along with radiofrequency, is among the new non-HTs proposed. 69 70 Only two clinical trials 63 64 on laser therapy were found, which showed that there was a significant increase in the sexual score after two laser applications.

Although pelvic floor muscle exercises have been widely discussed, only a single clinical trial has been found. The results concerning pelvic floor muscle showed that exercises have the potential to improve sexual function and are thus suggested to accompany other healthcare treatments designed for postmenopausal women. 59 60 A study 65 on the use of lidocaine in women with dyspareunia showed that it could enable comfortable intercourse; thus, it is a treatment that can be considered. Moreover, vitamin E vaginal suppository may be an alternative to vaginal estrogen in relieving the symptoms of vaginal atrophy in postmenopausal women, especially those not able to use HTs or those who have low compliance. 66

Ospemifene is a selective estrogen receptor modulator (SERM) that has been approved for the treatment of dyspareunia associated with VVA due to menopause. According to the studies 52 53 54 55 56 included in the present review, it was shown to be effective and well tolerated for the treatment of vaginal dryness and dyspareunia symptoms associated with VVA.

A wide variety of phytoestrogens are being used to improve sexual function in symptomatic postmenopausal women. The phytoestrogens found in the present review were as follows: *soy flour* , 32 *ginseng* , 33 62 *isoflavones* , 34 35 44 *C. foetida* , 30 *Glycine max* ( *L* .), 36 37 *T. terrestris* , 38 39 *fennel cream* , 40 *ginkgo biloba* , 41 and *Pueraria mirifica* . 42 Studies 38 39 have shown that *T. terrestris* could be a safe alternative for the treatment of sexual desire disorder, since its probable mechanism of action involves an increase in the serum levels of free and bioavailable testosterone.

Vaginal HTs, DHEA, and testosterone may have a positive impact on sexual desire and sexual function. The present review indicates that the vaginal HT with DHEA may have a positive impact on sexual desire and sexual function. This beneficial effect was apparent in all domains of sexual function, not only in those related to dyspareunia and vaginal dryness. 27 45 46 47 48 49 50 51 A meta-analysis conducted by Scheffers et al. 71 showed that oral and intravaginal DHEA used in peri- and postmenopausal women resulted in slight improvement in sexual function over placebo. Peixoto et al. 72 concluded that intravaginal DHEA is effective in improving several aspects of sexual function; however, there was no evaluation of vaginal dryness.

Regarding testosterone, what is known so far is that the efficacy of the therapy may rely on synergistic effects with estrogen. 16 19 57 58 Previous reviews have revealed controversial conclusions. A study conducted by Khera 73 demonstrated that systemic testosterone might improve sexual desire, arousal, pleasure, and overall satisfaction. The data obtained from studies by Pitsouni et al. 7 and Reed et al. 74 pointed out that, in cases of low sexual desire, testosterone is not indicated. The results obtained in the present review are in agreement with those of the literature, as there was no difference in sexuality scores and sexual satisfaction when comparing topical testosterone and estrogen levels.

Most treatments are considered safe, and no side effects were considered severe, only moderate to mild. It is important to highlight the lack of data about the side effects in some studies, which ends up generating uncertainties the true safety of treatment. According to the GRADE classification, the present study provided moderate or very low certainty of evidence that HTs or non-HTs are useful to improve sexual function in postmenopausal women. Consequently, the findings of the present study cannot be generalized until new randomized clinical trials are performed to confirm the strength of the evidence.

## Conclusion

The present systematic review identiﬁed that the literature lacks coherence in terms of the proposed treatments and selected outcome measures. There is great diversity and heterogeneity in the scales used for the assessment of sexual dysfunction, despite the fact that they have been validated, hence the need for standardization in the scores. One of the most commonly used, the Female Sexual Function Index, is problematic regarding non-sexually active women. The present review should be considered in the context of its limitations. Despite the great diversity in treatment modalities and outcome measures, the review can shed light on potential targets for treatment, which is deemed necessary for sexual dysfunction, assuming that most randomized trials were evaluated with a low risk of bias, according yours methodological characteristics.
