# Supplementary material for: Efficacy of Hormonal and Nonhormonal Approaches to Vaginal Atrophy and Sexual Dysfunctions in Postmenopausal Women: A Systematic Review
Source: Rev Bras Ginecol Obstet. 2022 Nov 29;44(10):986–94. doi: 10.1055/s-0042-1756148 (PMC9708399; doi:10.1055/s-0042-1756148)
Supplement: Supplementary file 1 — Supplementary Material [file 10-1055-s-0042-1756148-s200297.pdf]

Table 1 Characteristics of the studies included in the systematic review

Author (year)	Country	Design	Follow-up	Mean age (years)	N	Inclusion/ exclusion criteria	Interventions	Outcomes measured	Outcomes	Side effects	Certainty[a]
**Bygdeman and Swahn** (1996)[14]	Sweden	RCT	12 weeks	43–76	39	Inclusion criteria: vaginal dryness; age: 43-76. Exclusion criteria: hormonal-dependent cancer; HRT; thromboembolism; vaginal infection; and vaginal use of lubricant.	1. Dienoestrol; 2. replens.	Evaluated vaginal dryness index, pH, itching, irritation, dyspareunia, safety, AEs, and vaginal cytology.	Dyspareunia: difference between groups; vaginal dryness: in favor of dienoestrol after the first week of treatment.	Two AEs were reported, one in each group of treatment.	Very low ⊕ OOO
**Loprinzi et al.** (1997)[15]	USA	DBCO	4 weeks	18–65	45	Inclusion criteria: breast cancer and dryness; exclusion criteria: vaginal infection, HRT, pregnant or lactating, and use of any vaginal preparations.	1. Water-based lubricant-placebo; 2. polycarbophil-based.	Questionnaires asked patients how much vaginal dryness, itching, and discomfort during intercourse	Vaginal dryness: no differences between groups. Dyspareunia: improvement in symptoms	No serious toxicities were encountered.	Very low ⊕ OOO
**Balk et al.** (2002)[32]	USA	DBP RCT	6 months	56.8–57.9	27	Inclusion criteria: postmenopausal and with uterus. Exclusion criteria: tamoxifen usage, endometrial cancer, allergy to soy, HRT or supplementing with phytoetrogenic.	1. Placebo; 2. soy flour 100 mg/day.	Severity of menopause-associated symptoms was assessed using a questionnaire and suggested four-point scale.	Vaginal dryness: no differences between groups.	No other differences in symptoms or side effects.	Low ⊕⊕ OO
**Labrie et al.** (2009)[45]	USA and Canada	Phase-III DBP-M	12 weeks	42–74	216	Inclusion criteria: sexually active postmenopausal women, vaginal dryness, low maturation index, and vaginal pH ≥ 5.0. Exclusion criteria: hyperplasia /endometrial cancer, and HRT	1.DHEA-placebo; 2. 0.25%DHEA; 3. 0.5% DHEA; 4. 1.0% DHEA.	Changes in the sexual dysfunction parameters. The questionnaires used were the ASF, MENQOL and PGWB.	MENQOL: improvements in the desire domain. ASF: improved in the desire, lubrication, orgasm and dryness domain during intercourse.	–	High ⊕⊕⊕⊕
**Bachmann and Komi** (2010)[52]	USA	DBP RCT	12 weeks	58.4–58.9	826	Inclusion criteria: age between 40 and 80 years, FSH levels ≥ 40 IU/L, VVA: 5% or less superficial cells, pH ≥ 5.0. Exclusion criteria: endometrial thickness ≥ 4 mm or gynecological abnormalities, BMI ≥ 37 kg/m^2, vaginal creams, *digitalis* alkaloids or HRT.	1. Placebo; 2.ospemifene 30 mg; 3.ospemifene 60 mg.	Evaluated percentage of superficial and parabasal cells on the vaginal smear, vaginal pH, and self-assessed MBS of vaginal dryness or dyspareunia.	Vaginal dryness decreased in both ospemifene groups when compared to the placebo group. Dyspareunia for MBS decreased in the ospemifene 60 mg group compared with the placebo.	Ospemifene was shown to be safe and well tolerated. Hot flushes, a common AE associated with the use of SERMs, were reported.	Low ⊕⊕ OO
**Oh et al.** (2010)[33]	Korea	DBP RCT	8 weeks	47.1–55.3	32	Inclusion criteria: sexually-active menopausal women (more than one sexual intercourse /month) and FSH ≥ 40 mIU/mL. Exclusion criteria: hysterectomy, abnormal blood, HRT, and	1. Placebo; 2.ginseng 1g/day (KRG).	The FSFI and the GAQ were used to evaluate the effectiveness of the treatment on female sexual function.	GAQ: KRG -better effect than placebo. Desire, lubrication, orgasm and pain: no difference. An increase in the arousal domain. General score: no difference.	–	Low ⊕⊕ OO

(*Continued*)

 © 2022. Federação Brasileira de Ginecologia e Obstetrícia. All rights reserved.

Table 1 (*Continued*)

Author (year)	Country	Design	Follow-up	Mean age (years)	N	Inclusion/ exclusion criteria	Interventions	Outcomes measured	Outcomes	Side effects	Certainty[a]
						chemotherapy or pelvic radiation.					
**Raghunandan et al.** (2010)[16]	India	RCT	12 weeks	51.36–51.6	75	Inclusion criteria: postmenopausal women aged 40–65 years with urogenital and sexual dysfunction causing pain and affecting desire, arousal, or orgasm.<br>Exclusion criteria: contraindication to HRT and women using any hormonal treatment.	1. IVE: 0.625 mg of conjugated estrogen;<br>2. IVE: 0.625 mg of conjugated estrogen + 2% IVT 0.5 mg;<br>3. lubricant	Urogenital and sexuality scores. Vaginal health and maturation indices were calculated at the beginning and end of therapy.	McCoy FSQ: SS improvement in all groups. Advantage of combined IVE with IVT.	Testosterone therapy may result in side effects like hirsutism, acne, weight gain, voice changes, and clitoromegaly.	High ⊕⊕⊕⊕
**Ekin et al.** (2011)[67]	Turkey	RCT	8 weeks	51.86–52.95	42	Inclusion criteria: age ≥ 45 years, dryness and soreness. $E_2 \leq 20$ pg/mL and/or ≤ 5% superficial cells.<br>Exclusion criteria: hormone-dependent tumor, genital bleeding, vaginal infection, or any serious disease.	1. IVE 25 µg (estradiol);<br>2. Hyaluronic acid 5 mg.	Atrophic vaginitis was evaluated by vaginal score: determination of vaginal health by examination of epithelial atrophy, vaginal pH and vaginal cytology.	Dyspareunia: disappearance of severe intensity.<br>Vaginal dryness: disappearance of severe intensity.	–	Very low ⊕ OOO
**Genazzani et al.** (2011)[46]	Italy	RCT	12 months	53.5–55.2	48	Inclusion criteria: menopausal women aged 50–60 years.<br>Exclusion criteria: endocrine and cardiovascular diseases, psychiatric disorders, HRT, smoking, presence of any kind of pelvic and breast disease.	1. DHEA 10mg/day;<br>2. oral estradiol 1 mg/day + dihydrogesterone 5 mg/day;<br>3. oral tibolone 2.5 mg/day;<br>4. oral vitamin D 400 IU + calcium carbonate 1,250 mg	The self-administered questionnaire used was the McCoy FSQ.	McCoy FSQ: the groups receiving DHEA or HRT reported improvement in sexual function compared to baseline using the McCoy total score.	All the patients who enrolled in the study completed the follow-up, without any AEs.	Very low ⊕ OOO
**Jonasson et al.** (2011)[61]	Sweden	DBP RCT	7 days	59.7–60.4	20	Inclusion criteria: postmenopausal women with symptoms of vaginal atrophy who had not used any estrogen or other hormonal treatments.<br>Exclusion criteria: endocrine disease and other serious illnesses.	1.Placebo<br>2. oxytocin.	Gynaecological examination and colposcopic inspection were performed. In addition, the patients filled out a questionnaire regarding symptoms.	VVA symptoms: improvement in both groups. No SS difference between groups.	None of the study participants reported any side-effects.	Low ⊕⊕ OO
**Labrie et al.** (2011)[27]	USA and Canada	Phase-III DBP-M	12 weeks	42–74	216	Inclusion criteria: sexually-active menopausal women with vaginal atrophy, low maturation index, pH ≥ 5.<br>Exclusion criteria: abnormal bleeding, previous	1. DHEA-placebo;<br>2. 0.25% DHEA;<br>3. 0.5% DHEA;<br>4. 1.0% DHEA;	Dyspareunia, vaginal cell maturation, and pH were evaluated. The degree of the dyspareunia was classified as none, mild, moderate, or severe.	Dyspareunia: decrease in all groups. SS advantage of each DHEA group over placebo.	No adverse effect was observed on laboratory parameters.	Very low ⊕ OOO

 © 2022. Federação Brasileira de Ginecologia e Obstetrícia. All rights reserved.

Table 1 (*Continued*)

Author (year)	Country	Design	Follow-up	Mean age (years)	N	Inclusion/ exclusion criteria	Interventions	Outcomes measured	Outcomes	Side effects	Certainty[a]
						diagnosis of cancer, endometrial hyperplasia, and HRT.					
**Le Donne et al.** (2011)[28]	Italy	DB	3 months	58.3–49.4	62	Inclusion criteria: postmenopause symptomatic and vaginal atrophy. Exclusion criteria: atypical cervical vaginal smears, use of HRT.	1.Genistein 97 µg; 2. hyaluronic acid 5 mg.	Questionnaires asked how much vaginal dryness and degree of dyspareunia. Beyond the colposcopic features and vaginal smear.	Genital symptoms: decrease in both groups. Advantage of genistein.	There were no side effects.	High ⊕⊕⊕⊕
**Lee et al.** (2011)[17]	South Korea	DBP RCT	12 weeks	44.98–45.86	98	Inclusion criteria: breast cancer and vaginal dryness. Exclusion criteria: natural menopause before the diagnosis of breast cancer, severe medical diseases, or malignancies.	1. Placebo; 2. pH-balanced gel.	VHI, vaginal pH assessed from the lateral vaginal wall, and vaginal maturation index.	Dyspareunia: decrease in both groups. Vaginal dryness: decrease in both groups.	All AEs were considered mild in severity and self-limited.	Low ⊕⊕ OO
**Loprinzi et al.** (2011)[18]	USA	DBP2 RCT	6 weeks	54.6–55.1	201	Inclusion criteria: postmenopausal women with breast cancer or who did not take vaginal estrogen and had vaginal symptoms. Exclusion criteria: vaginal infection or vulvar/vaginal diseases, no use of vaginal preparations or HRT.	1. Placebo; 2. pilocarpine 5 mg twice a day; 3. pilocarpine 5 mg 4 times a day.	Vaginal dryness measured by vaginal pH, improvement in cytology index, improvement in vaginal atrophy symptoms.	The comparison of vaginal dryness symptoms in the collective pilocarpine arms against the placebo arm did not reveal any benefit of the pilocarpine.	Nausea, sweating, rigors and urinary frequency in the pilocarpine treatment.	Low ⊕⊕ OO
**Amato et al.** (2013)[34]	USA	DBP-M RCT	2 years	54.5–55.0	406	Inclusion criteria: postmenopausal with a FSH ≥ 30 mIU/mL. Exclusion criteria: any abnormalities on mammogram, Pap smear, or blood chemistry, BMI ≥ 30 kg/m^2, and any HRT.	1. Placebo; 2. isoflavones 80 mg/day; 3. isoflavones 120 mg/day.	Quality of life was assessed using the MENQOL questionnaire administered at baseline, 1 year, and 2 years.	MENQOL –sexual domain: no differences between groups.	One woman in the soy isoflavones 120mg group was found with breast cancer and one in the isoflavones 80 mg, with endometrial cancer.	Low ⊕⊕ OO
**Grimaldi et al.** (2012)[29]	Italy	DBP	3 days	57.4	36	Inclusion criteria: postmenopausal women. Exclusion criteria: women who were taking any vaginal hormone (other than thyroxine) or had vaginal infection.	1. Placebo; 2. hyaluronic acid.	At the end, patients underwent a final examination to evaluate the symptoms and the presence of AEs.	Vaginal dryness: Decreased in both groups. However, no difference statistic between groups.	No AEs occurred during the entire period of the study.	Low ⊕⊕ OO
**Tedeschi and Benvenuti** (2012)[35]	Canada	M RCT	4 weeks	53.4–53.9	186	Inclusion criteria: postmenopausal women with vaginal dystrophy.	1. No treatment; 2. isoflavones 10 mg.	The severity the of symptoms was scored, and pH was measured.	Dyspareunia: decrease in the isoflavones group.	Events associated with estrogens: bleeding,	Very low ⊕ OOO

(*Continued*)

 © 2022. Federação Brasileira de Ginecologia e Obstetrícia. All rights reserved.

Table 1 (*Continued*)

Author (year)	Country	Design	Follow-up	Mean age (years)	N	Inclusion/ exclusion criteria	Interventions	Outcomes measured	Outcomes	Side effects	Certainty[a]
									Vaginal dryness: decrease in the isoflavones group.	pelvic pain and breast tenderness.	
**Chen et al.** (2013)[68]	China	OLP-M RCT	30 days	54.4–54.05	144	Inclusion criteria: age ≤ 70 years, with vaginal dryness, and no contraindications to HRT. Exclusion criteria: unmarried, vaginal infections, estrogen hormone-dependent tumors, and vaginal products.	1. IVE (estriol) 0.5 g; 2. hyaluronic acid 5 g.	Efficacy was assessed by questionnaire to evaluate vaginal dryness and dyspareunia during a phone contact after the third administratio/end of treatment.	Dyspareunia: o difference between groups. Vaginal dryness: advantage of estrogens but no significant difference regarding hyaluronic acid.	13 AEs: 7 in the test group (3 had a suspicious relationship with the test product), and 6 AEs in the control group.	Very low ⊕ ooo
**Lima et al.** (2013)[36]	Brazil	DBP RCT	12 weeks	56–57	90	Inclusion criteria: menopausal women, vaginal atrophy, E_2 levels ≤ 20 pg/mL, FSH ≥ 40 mIU/mL, no superficial cells, and endometrial thickness ≤ 4.0 mm. Exclusion criteria: HRT, corticosteroids, hormone-dependent tumor.	1. Placebo 1 g; 2. isoflavones 1 g; 3. conjugated equine estrogens 0.625 mg (CEE).	Symptoms of vaginal dryness and dyspareunia, which were reported and classified as none, mild, moderate and severe. Vaginal smears were taken to determine the MI.	Dyspareunia: decrease in all groups. No difference between isoflavones and CEE. Vaginal dryness: decrease in all groups. Advantage of CEE over isoflavones.	No serious AEs occurred in the study.	High ⊕⊕⊕⊕
**Portman et al.** (2013)[53]	USA	DBP-M RCT	12 weeks	58.0–58.1	605	Inclusion criteria: age 40 to 80 years, FSH ≥ 940 IU/L, superficial cells ≤ 5%, and a vaginal pH ≥ 5. Exclusion criteria: BMI ≥ 37 kg/m^2 or unknown uterine bleeding, uterine diseases, vaginal infection, and abnormal findings.	1. Placebo; 2. ospemifene 60 mg/day.	Percentage of parabasal cells and percentage of superficial cells in the MI of the vaginal smear, vaginal pH, and severity of the MBS.	Dyspareunia: significantly reduced versus placebo.	The number of participants who withdrew from the study because of an adverse effect was higher in the ospemifene group.	Low ⊕⊕ oo
**Zheng et al.** (2013)[30]	China	RCT	3 months	52.1–53.4	96	Inclusion criteria: amenorrhea ≤ 6 months and within 5 years, $E_2 < 30$ pg/ml, and FSH > 40 IU/L. Exclusion criteria: uterine diseases, severe diseases, breast cancer, HRT, and endometrial thickness ≥ 0.5	1. *Cimicifuga foetida*/ daily; 2. estradiol valerate + progesterone capsule; 3. estradiol valerate + medroxyprogest.	MENQOL was applied. The severity of each symptom was assessed on a 7-point scale, from minor to intense.	MENQOL score: no significant difference was observed in scores of each domain among the 3 groups before treatment.	Breast tenderness and vaginal bleeding were lower in the *cimifuga* group than the HRT groups.	Very low ⊕ ooo
**Constantine et al.** (2015)[54]	USA	DBP-M RCT	12 weeks	58.5–58.7	919	Inclusion criteria: postmenopausal women with VVA. Exclusion criteria: none.	1. Placebo; 2. ospemifene 60 mg/day	Participants filled out the FSFI at baseline, 4 and 12 weeks, and the scores were calculated	FSFI total score: improvement in scores. FSFI domains: improvements in all domains.	The number of women who withdrew from the study was similar in both groups	Very low ⊕ ooo
	Brazil	RCT	12 weeks	56.2–57.7	80						Low ⊕⊕ oo

 © 2022. Federação Brasileira de Ginecologia e Obstetrícia. All rights reserved.

Table 1 (*Continued*)

Author (year)	Country	Design	Follow-up	Mean age (years)	N	Inclusion/ exclusion criteria	Interventions	Outcomes measured	Outcomes	Side effects	Certainty[a]
**Fernandes et al.** (2014)[19]						Inclusion criteria: physiological postmenopause women aged 40–70 years with vaginal atrophy. Exclusion criteria: women with BMI < 18.5 kg/m^2 or > 30 kg/m^2, contraindication to HRT, and positive serology for HIV and hepatitis B or C.	1. Polyacrilic acid 3 g/day; 2. IVT 0.3 mg/day; 3. IVE 0.625 mg/day; 4.glycerin lubricant 3 g/day.	FSFI was applied and individual scores were obtained by adding up the items that comprise each domain. The total score was obtained.	FSFI: desire/pain: improvement; excitation: improvement for IVT; lubrication: improvement for all groups; orgasm/satisfaction: improvement for IVT.	Women who used topical testosterone did not show androgenic side effects such as acne, increased hair growth, and clitoral hypertrophy.	
**Labrie et al.** (2014)[47]	USA and Canada	Phase-III DBP-M	12 weeks	42–74	216	Inclusion criteria: symptomatic menopausal women, low maturation index, and vaginal pH ≥ 5. Exclusion criteria: abnormal bleeding, previous diagnosis of cancer, endometrial hyperplasia, use of HRT.	1. DHEA- placebo; 2. 0.25% DHEA; 3. 0.5% DHEA; 4. 1.0% DHEA.	Desire, arousal and orgasm were self-rated by the women at screening using the ASF and MENQOL questionnaires.	MENQOL: desire/dryness – improvement in all groups. ASF: desire and lubrication domain improvement in all groups. Orgasm domain increase only in the DHEA groups.	–	Very low ⊕ ०००
**Lima et al.** (2014)[37]	Brazil	DBP RCT	12 weeks	59.9–59.2	60	Inclusion criteria: age ≥ 45 years, vaginal dryness, and sexual activity, E_2 ≤ 20 pg/mL, FSH ≥ 40 mIU/mL, no superficial cells on vaginal cytology. Exclusion criteria: HRT, hormone-dependent tumor, thromboembolic disorder, and vaginal infection.	1. Placebo 1 g; 2. Isoflavones 1 g.	Patients reported vaginal dryness, pruritus, pain/soreness, vulvar or vaginal burning and dyspareunia, which were classified as follows: none, mild, moderate, severe.	Dyspareunia: SS decrease in isoflavones group. Advantage of isoflavones (SS). Vaginal dryness: SS decrease in both groups. No difference between groups.	No serious AEs occurred in the study.	High ⊕⊕⊕⊕
**Portman et al.** (2014)[55]	USA	DBP-M RCT	12 weeks	59.3–59.8	314	Inclusion criteria: VVA was assessed by the maturation index of the vaginal smear (≤ 5% superficial cells) and vaginal pH (> 5). Exclusion criteria: BMI ≥ 37 kg/m^2, abnormal gynecological findings, and any HRT.	1. Placebo; 2. ospemifene 60 mg/day.	The primary endpoints were the percentage of parabasal and superficial cells, vaginal pH, and vaginal dryness. The secondary endpoints were sexual activity and lubricant.	Vaginal dryness: change in severity score from baseline reported by women receiving ospemifene compared with placebo.	The most reported adverse effects in the ospemifene group were urinary tract infection, hot flushes, and pharyngitis.	Very low ⊕ ०००
**Archer et al.** (2015)[48]	USA and Canada	Phase-III DBP-M	12 weeks	57.51–59.37	255	Inclusion criteria: Women hysterectomized or not, with FSH ≥ 40 IU/L, ≤ 5% of superficial cells on vaginal epithelium, and vaginal pH ≥ 5.	1. DHEA-placebo; 2. 0.25% DHEA; 3. 0.5% DHEA.	Vaginal atrophy symptoms were categorized as none, mild, moderate, or severe. Vaginal dryness and itching were evaluated by a questionnaire.	Dyspareunia: decrease in all groups. Vaginal dryness: SS decrease in all groups.	Based on the investigators' assessment, frequencies of adverse effects ranged from 3.4% (0.50% DHEA) to 5.0% (placebo).	Very low ⊕ ०००

(*Continued*)

 © 2022. Federação Brasileira de Ginecologia e Obstetrícia. All rights reserved.

Table 1 (*Continued*)

Author (year)	Country	Design	Follow-up	Mean age (years)	N	Inclusion/ exclusion criteria	Interventions	Outcomes measured	Outcomes	Side effects	Certainty[a]
**Bouchard et al.** (2015)[49]	USA and Canada	Phase-III DBP-M	6 and 12 weeks	57.59–58.41	450	Inclusion criteria: Non hysterectomized, FSH ≥ 40 IU/L, aged 40–75 years, with 5% or fewer superficial cells, vaginal pH >5, and normal mammogram and Pap smear results. Exclusion criteria: cancer, any HRT.	1. DHEA-placebo; 2. 0.25% DHEA; 3. 0.5% DHEA.	Vaginal atrophy was recorded as none, mild, moderate or severe, dryness, was evaluated, as well as dyspareunia and vulvovaginal itching, as a secondary objective.	Dyspareunia: SS decrease in all groups up to 12 weeks. At 12 weeks, SS difference between groups was lost. Dryness: SS decrease in all groups up to 12 weeks. At 12 weeks, SS between groups was lost.	Half of the subjects experienced at least one adverse effect.	Very low ⊕ ooo
**Goetsch et al.** (2015)[65]	USA	DBP RCT	12 weeks	54.0–56.6	46	Inclusion criteria: a stable heterosexual relationship, not using estrogen products for at least 4 months, speaking English, and age between 18 and 70 years. Exclusion criteria: dyspareunia, pelvic pain, pelvic floor myalgia, or vulvar dermatoses.	1. Placebo; 2.aqueous lidocaine 4%.	The primary outcome was pain with intercourse, scored by using the Numeric Rating Scale. The secondary outcomes were resumption of intercourse and improvement in sexual function.	Dyspareunia: decrease in the SFQ score. Desire and arousal: improvement in the lidocaine group. Orgasm: No improvement. Pain: improvement in the lidocaine group.	–	Low ⊕⊕ oo
**Labrie et al.** (2015)[50]	USA	Phase-III DBP	6 and 12 weeks	59.5–59.6	554	Inclusion criteria: vulvovaginal atrophy symptoms. Exclusion criteria: fibroids and endometrial polyps, any RHT.	1. DHEA-placebo; 2. 0.50% DHEA.	The FSFI questionnaire was filled out during screening visit, and at day 1, week 6, and week 12.	FSFI: desire, arousal, orgasm, lubrication, and satisfaction: increase in both groups. Pain: Improved in both groups.	–	Very low ⊕ ooo
**Tungmunsakulchai et al.** (2015)[57]	Thailand	DBPC RCT	8 weeks	52.7–53.8	70	Inclusion criteria: age 40–60 years, sexually-active, diagnosed with sexual dysfunction, FSFI scores ≤ 26.5 and literate. Exclusion criteria: severe vasomotor symptoms, cerebrovascular or cardiovascular disease, cancer, psychiatric disorders, HT or any psychiatric drugs in the past 3 months.	1.Oral testosterone undecanoate 40 mg + oral estrogen daily 2. placebo + oral estrogen daily.	The FSFI scores before and after treatment were compared to assess any improvement in sexual function.	After 8 weeks of treatment, the FSFI scores significantly improved in both groups when compared to the baseline scores, but the FSFI scores from the testosterone group were higher than those of the placebo group posttreatment.	There was no serious adverse effect. The participants reported having mild acne and mild facial hair growth, which were comparable between both groups.	Low ⊕⊕ oo
**Hickey et al.** (2016)[20]	Australia	DBCO RCT	4 weeks	44.7–61.5	46	Inclusion criteria: vaginal dryness or pain during sexual activity, willingness to keep a sexual activity diary, and normal Pap smear. Exclusion criteria: use of steroids, vaginal infection, and clinically	1.Water-based lubricant (placebo); 2.Silicone-based lubricant.	SAQ, FSDS-R, FIEI. Completed sexual activity diaries assessing characteristics of lubricant application and discomfort. On the final visit, patients designed preference item.	Dyspareunia: improvement during use of silicone lubricant. FIEI: lubrication/ability to orgasm: increased. SAQ and FSDS-R: No SS difference between groups.	One patient discontinued the intervention because of a treatment-related AE (vulval itching).	Low ⊕⊕ oo

 © 2022. Federação Brasileira de Ginecologia e Obstetrícia. All rights reserved.

Table 1 (*Continued*)

Author (year)	Country	Design	Follow-up	Mean age (years)	N	Inclusion/ exclusion criteria	Interventions	Outcomes measured	Outcomes	Side effects	Certainty[a]
						significant anxiety or depression.					
**Jokar et al.** (2016)[31]	Iran	RCT	8 weeks	51.92–56.4	100	Inclusion criteria: moderate to severe dryness, endometrial thickening ≤ 5 mm. Exclusion criteria: use of anti-coagulant drugs, topical hormonal/ nonhormonal drugs, and vaginal infection.	1. Conjugated estrogens cream 0.625 mg; 2. hyaluronic acid 5 mg.	The severity of the atrophy was evaluated by VAS before and after the intervention and based on a four-point scale ranging from asymptomatic to severe.	VAS: vaginal dryness, itching, and dyspareunia significantly improved in all groups after the intervention. among the groups.	Hyaluronic acid was tolerated well, without side-effects.	High ⊕⊕⊕⊕
**Juliato et al.** (2017)[21]	Brazil	RCT	30 days	48.8–50.5	60	Inclusion criteria: use of tamoxifen for breast cancer patients who complained of GSM and sexual intercourse. Exclusion criteria: patients who were unable to understand the questionnaires.	1. Lubricant; 2. polyacrilic acid.	The patients answered one questionnaire about pain and discomfort during sexual intercourse and the FSFI.	Dyspareunia and vaginal dryness: o difference between groups. FSFI: no difference between groups.	The adverse effects were hot flushes, vaginal dryness, insomnia, weight gain, irritability, and depression or mood swings.	Very low ⊕ ooo
Labrie et al. (2016)[51]	USA	Phase-III DBP	12 weeks	59.5–59.6	482	Inclusion criteria: post menopause, between 40–80 years, FSH levels > 40 IU/L, pain during sexual activity; superficial cells < 5%, and vaginal pH ≥ 5. Exclusion criteria: cancer and any HRT.	1. DHEA-placebo; 2. 0.5% DHEA.	The symptom score was classified as none, mild, moderate or severe. The aspect of the mucosa was verified by gynecological examinations.	Dyspareunia: SS decrease in both groups. Vaginal Dryness: SS decrease in both groups.	Adverse effects were found in 47.9% of the DHEA group. The most frequent side effect was infection.	Low ⊕⊕ oo
**Melisko et al.** (2017)[58]	USA	Phase-II RCT	12 weeks	56–57	76	Inclusion criteria: dryness, dyspareunia, or decreased libido, and $E_2 \leq$ 10 pg/mL. Exclusion criteria: history of vaginal or vulvar radiation, gynecologic cancer, abnormal Pap smear within 1 year, or any HRT.	1. IVE 2 mg 2. IVT 0.5 mg d for 2 weeks; then, 0.5 mg 3 times/week for 10 weeks.	The vaginal epithelium was assessed using a 4-point scale evaluating rugae, pallor, petechiae, elasticity, and dryness. Patients filled out the CARES.	Sexual interest and sexual dysfunction: SS improvement in both groups. 0.1-unit advantage for IVT. Sexual satisfaction score: increase in both groups (SS only for IVE).	Adverse effects: vaginal discharge, facial hair growth, vaginal/vulvar itching, odor and/or urinary infection.	High ⊕⊕⊕⊕
**Postigo et al.** (2016)[38]	Brazil	DBP RCT	3 months	45–47	74	Inclusion criteria: 1 year of amenorrhea and FSH levels > 30 mUI/mL, sexually active, stable partner, and sexual dysfunction. Exclusion criteria: any HRT, diabetes mellitus, hormone-dependent tumor, and severe diseases.	1. Placebo; *2.T. Tribulus terrestris*.	The questionnaires used in the sex interview, with the purpose of obtaining epidemiological data, were the SFQ and FIEI.	SFQ: differences between the groups in the desire, sexual interest, arousal and interaction domains. FIEI: improvement in vaginal foreplay, and the ability to reach orgasm.	The most frequent were diarrhea, nervousness, dizziness and nausea in the Tribulus group, and nervousness, facial flushing, dizziness, and nausea in the placebo group.	Low ⊕⊕ oo

(*Continued*)

 © 2022. Federação Brasileira de Ginecologia e Obstetrícia. All rights reserved.

Table 1 (*Continued*)

Author (year)	Country	Design	Follow-up	Mean age (years)	N	Inclusion/ exclusion criteria	Interventions	Outcomes measured	Outcomes	Side effects	Certainty[a]
**Seyyedi et al. (2016)**[22]	Iran	DB RCT	3 months	52,79–54.37	90	Exclusion criteria: grade-2 prolapse, bladder and uterus sexual problems, migraine, use of hormones, and sensitivity to use of estrogenic.	1. Lubricant<br>2. premarin<br>3. royal jelly.	The MENQOL questionnaire evaluated quality of life, vasomotor problems, psychosocial problems, and physical and sexual problems.	MENQOL – sexual domain: no difference between premarin and royal jelly.	–	Low ⊕⊕ oo
**De Souza et al.** (2016)[39]	Brazil	DBP RCT	120 days	43–65	45	Inclusion criteria: FSH $\geq$ 30 IU/L, estradiol $\leq$ 40 pg/mL, and BMI $\leq$ 28.<br>Exclusion criteria: relationship problems or use of HTR or any drugs that interfere with sexual desire, breast or endometrial cancer, and previous oophorectomy.	1. Placebo;<br>2. *T. terrestris* 750 mg/dat	All the selected participants answered the FSFI and the SFQ questionnaires.	FSFI: improvements in the general score.<br>SFQ: improvementsin sexual desire, arousal and lubrication, pain. and orgasm.	*T. terrestris* seems to be safe, only six women withdrew from the study due to mild side effects.	Low ⊕⊕ oo
**Yaralizadeh et al.** (2016)[40]	Iran	DBPC RCT	8 weeks	52.9–53.7	60	Inclusion criteria: natural menopause, elevation of FSH and LH, atrophy, and sexual activity.<br>Exclusion criteria: vaginal infection, hormone use, smoking, alcohol use, uterine bleeding of unknown cause, and use of phytoestrogen	1. Placebo;<br>2. fennel cream.	To evaluate vaginal atrophy, each participant was asked if they had symptoms such as vaginal dryness, vaginal itching, burning, and dyspareunia.	Dyspareunia: SS improvement in the fennel group.<br>Vaginal dryness: SS improvement in the fennel group.	No adverse effects were reported during the study period in either group.	Low ⊕⊕ oo
**Cruz et al.** (2018)[63]	Brazil	DBP RCT	8 and 20 weeks	55.7–55.9	45	Inclusion criteria: age 45–70 years, amenorrhea for 24 months, and at least 1 symptom of VVA.<br>Exclusion criteria: BMI $\geq$ 35kg/m^2, drug-induced menopause, cancer, vaginal radiotherapy, vaginal creams, or HRT.	1. CO_2 laser + placebo estriol (L group);<br>2. CO_2 laser + estriol (LE group);<br>3. sham CO_2 laser + estriol (E group).	Both VHI and VVA were assessed at weeks 0, 8 and 20. Participants rated VVA symptoms. Secondary outcome: analysis of vaginal smear samples and sexual function (FSFI).	The VHI average score was SS higher in all arms. The L and LE groups showed a SS improvement in dyspareunia, burning, and dryness.<br>The L group showed SS worsening in the pain domain of the FSFI; however, the FSFI total scores were comparable in all treatment arms at week 20.	–	High ⊕⊕⊕⊕
**Malakouti et al.** (2017)[41]	Iran	DB RCT	6 weeks	48.8–51.8	180	Inclusion criteria: women with natural menopause, not taking any effective drug on sexual response, lack of mental health problems or systemic diseases affecting sexual	1. Placebo;<br>2. *Ginkgo biloba* tablets 40; mg<br>3. 2-3 drops of aromatic solution.	The FSFI questionnaire was filled out before and after the intervention.	FSFI general score: differences among the three groups.	–	Low ⊕⊕ oo

 © 2022. Federação Brasileira de Ginecologia e Obstetrícia. All rights reserved.

Table 1 (*Continued*)

Author (year)	Country	Design	Follow-up	Mean age (years)	N	Inclusion/ exclusion criteria	Interventions	Outcomes measured	Outcomes	Side effects	Certainty[a]
						function. Exclusion criteria: none.					
**Nappi et al.** (2017)[23]	Italy	OLP-M RCT	8 weeks	56.8–56.5	95	Inclusion criteria: menopausal women with at least 2 years of amenorrhea, dryness, and normal endometrium.<br>Exclusion criteria: perimenopausal, genitourinary infections, prolapse, use of any HTR.	1. No treatment;<br>2.Monurelle biogel.	A VRS was self-administrated by daily record card. Secondary endpoints: differences from baseline to the end of the study in: VRS, VHI, MI, FSFI, FSDS-R.	Advantage for Monurelle biogel. Improved FSDS-R score, FSFI score, VVA symptoms and vaginal dryness.	Four AEs: burn, discomfort, vulvovaginal pruritus, and pruritus.	Very low ⊕ ooo
**Nazarpour et al.** (2017)[59]	Iran	RCT	12 weeks	51.5–53.1	145	Inclusion criteria: natural menopause within 3 years, sexually active, no cardiovascular or mental disorders, absence of psychological distress, no history of drug addiction, not undergoing any chemical or herbal HT.<br>Exclusion criteria: debilitating diseases, experiencing marital discord, getting divorced, and death or illness of the husband during the study period.	1. Formal sex education;<br>2. Kegel exercises; 3. routine care.	Sexual function was assessed using the FSFI.	FSFI score: no SS among the three study groups at the outset of the study. Scores of arousal, orgasm and satisfaction in the formal sex education and Kegel groups were significantly higher compared with the control group, after 12 weeks.	–	Low ⊕⊕ oo
**Suwanvesh et al.** (2017)[42]	Thailand	RCT	12 weeks	56.4–55.7	82	Inclusion criteria: intact uterus and last menstrual period at least 1 year before, no HTR and no contraindication to HTR.<br>Exclusion criteria: contraindication to HTR, breast or genital cancer.	1.*Pueraria mirifica* gel;<br>2. conjugated equine estrogens 0.625 mg.	A questionnaire evaluated dryness, soreness, irritation, dyspareunia, and vaginal discharge and pelvic examinations.	Dispareunia: diminuição observada em ambos os grupos. No entanto, não houve diferença estatística entre os grupos.<br>Vaginal dryness: decrease in both groups.	–	Low ⊕⊕ oo
**Diem et al.** (2018)[24]	USA	DBPC RCT	4 and 12 weeks	61	302	Inclusion criteria: age 45–70 years, 2 or more years since last menses, reported 1 or more moderate–severe symptom (s): vulvovaginal itching, pain, dryness, irritation, or pain.<br>Exclusion criteria: current vaginal infection; use of HRT, antibiotics, vaginal moisturizer, douche, and	1. Estradiol tablet plus placebo gel (10 μg);<br>2. vaginal moisturizer plus<br>placebo tablet;<br>3. dual placebo.	Sexual function was evaluated the four MENQOL.	A SS group mean difference favoring vaginal estradiol was observed for the MENQOL sexual function domain, but not for any of the other domains.	–	Low ⊕⊕ oo

(*Continued*)

 © 2022. Federação Brasileira de Ginecologia e Obstetrícia. All rights reserved.

Table 1 (*Continued*)

Author (year)	Country	Design	Follow-up	Mean age (years)	N	Inclusion/ exclusion criteria	Interventions	Outcomes measured	Outcomes	Side effects	Certainty[a]
						chronic premenopausal vulvovaginal symptoms.					
**Golmakani et al.** (2019)[66]	Iran	RCT	4, 8, and 12 weeks	30.8–38.5	52	Inclusion criteria: women aged 40–65 years, who were married and having sexual intercourse, who had had amenorrhea for at least 12 months and an FSH serum level > 40 IU, who had had a normal Pap smear in the last 3 years, with vaginal atrophy. Exclusion criteria: endometrial or breast cancer, vaginal bleeding, diabetes mellitus, kidney disease, arthritis, cardiovascular disease, vaginal infection, allergy to estrogen or HTR, and sexual dysfunction.	1. Vitamin E vaginal suppository; 2. conjugated estrogen vaginal cream.	The ASFQ was used as the primary outcome measure.	Overall scores of the ASFQ were increased significantly in both groups during the course of the study, compared with baseline ($p < 0.001$). However, the mean ASFQ scores of the two treatment groups did not differ significantly.	–	Very low ⊕ OOO
**Mitchell et al.** (2018)[25]	USA	DBP RCT	12 weeks	55–64	302	Inclusion criteria: 2 years since last menses, at least 1 symptom (itching, pain, irritation, dryness or pain). Exclusion criteria: vaginal infection, use of HTR, antibiotics or vaginal moisturizer in past month, and vaginal symptoms.	1. Vaginal estradiol + placebo gel; 2. vaginal moisturizer + placebo tablet; 3. dual placebo.	MBS was defined by itching, pain, dryness, irritation, or pain with penetration. Severity was rated 0 to 3. Secondary outcomes: VSI, pH and FSFI/FSDS–R.	FSFI score: Mean total improvement was similar between estradiol and placebo groups and between moisturizer and placebogroups.	Candidiasis diagnosed in 5 participants randomized to estradiol, 2, to moisturizer, and 2, to dual placebo.	Low ⊕⊕ OO
**Nazarpour et al.** (2018)[60]	Iran	RCT	12 weeks	52.84–53.13	104	Inclusion criteria: natural menopause, sexually active, absence of sexual dysfunction. Exclusion criteria: premature menopause, marital discord, and death or illness of spouse.	1. Educational programs; 2. PFM exercises.	FSFI questionnaire and a researcher-made questionnaire on personal and demographic information.	FSFI: excitation, orgasm, and satisfaction: differences between the 2 groups. General score: no differences.	–	Low ⊕⊕ OO
**Torky et al.** (2018)[62]	Egypt	MPRC RCT	30 days	54.1–54.58	140	Inclusion criteria: sexually-active menopausal women, vaginal atrophy, and no use of any HRT. Exclusion criteria: any serious illnesses or malignancy.	1. Placebo; 2. oxytocin 400UI.	Symptoms, histopathological evaluation of the vaginal mucosa, and estradiol level before and after treatment.	Dyspareunia: the scores showed a highly significant difference between the two groups after treatment.	No side effects from the gel were reported by any of the patients in the study.	Low ⊕⊕ OO
	USA		12 weeks	40–80	631						Low ⊕⊕ OO

 © 2022. Federação Brasileira de Ginecologia e Obstetrícia. All rights reserved.

Table 1 (*Continued*)

Author (year)	Country	Design	Follow-up	Mean age (years)	N	Inclusion/ exclusion criteria	Interventions	Outcomes measured	Outcomes	Side effects	Certainty[a]
**Archer et al.** (2019)[56]		DBP-M RCT				Inclusion criteria: postmenopausal, and/or FSH > 40 IU/L, vaginal dryness as MBS ≤ 5% of superficial cells on vaginal smear, and pH ≥ 5.0. Exclusion criteria: endometrial thickness ≥ 4 mm clinical abnormality, BMI ≥ 38 kg/m^2, and any HRT.	1. Placebo; 2. ospemifene 60 mg/day.	The four co-primary efficacy endpoints were changes in the percentages of parabasal cells and superficial cells, vaginal pH, and severity of the self-reported MBS of vaginal dryness.	Ospemifene improved vaginal dryness and dyspareunia. Secondary endpoints: FSFI improved with ospemifene versus placebo at week 12.	No unexpected TEAEs, treatment-related serious TEAEs, thrombotic events, or endometrial hyperplasia or carcinoma were observed.	
**Ghorbani et al.** (2019)[43]	Iran	DBPC RCT	4 weeks	52.9–53.5	62	Inclusion criteria: married postmenopausal women aged 45–60 years, sexually active, last menstruation occurred a minimum of 12 months ago and a maximum of 10 years ago, and FSFI score < 28 in the initial evaluation. Exclusion criteria: sexual dysfunction related to psychiatric disorder, HTR, cardiovascular disorders, diabetes/hypertension, low pressure, radical hysterectomy, and external genitalia deformity.	1. *Panax ginseng* (500 mg); 2. placebo.	Standard questionnaires, including the FSFI, the MENQOL, and the Greene Menopausal Symptom Scale, were completed before and four weeks after the intervention.	After the intervention, the mean total score of FSFI was significantly higher in the intervention group compared to the control group. The mean total score of menopausal symptoms was significantly lower in the treatment group than in the control group.	No side-effects mandating the discontinuation of the medication regimen were reported in this study.	High ⊕⊕⊕⊕
**Mitchell et al.** (2019)[26]	USA	DB RCT	12 weeks	45–70	302	Inclusion criteria: age 45-70 years, at least 1 vaginal symptom, and pain associated with sexual activity. Exclusion criteria: abnormal genital bleeding, pregnancy, acute vaginal infection, pelvic or vaginal surgery in previous 60 days, antibiotic use, breast or endometrial cancer.	1. Estradiol tablet 10 µg; 2. vaginal moisturizer; 3. placebo.	Proportion of sexually-active women at 12 weeks, frequency of sexual activity, and pain severity with sexual activity (0-3 scale).	Mean (standard deviation) pain with sexual activity scores at 12 weeks were similar between vaginal the estrogen (1.0 [1.0]) and placebo (0.9 [0.9], $p = 0.52$) groups.	–	Low ⊕⊕ OO
**Palma et al.** (2019)[44]	Italy	RCT	12 weeks and 3 months.	51.2–54.8	75	Inclusion criteria: age > 45 years, in physiological postmenopause (12 months of amenorrhea or 6 months of amenorrhea; FSH > 40IU/L). Exclusion criteria: contraindications to HT, cancer	1. Oral HT (conjugated equine estrogens 0.3 mg + medroxyprogesterone acetate 1.5 mg); 2. oral soy isoflavones (75 mg twice a day); 3.	The sexual function was evaluated by the MenQoL scale.	The MenQoL score decreased similarly in the three treatment groups. The sexual subscale declined significantly only during acupuncture ($p < .05$).	No serious adverse effect was reported. Only three women in the acupuncture group reported at the first session a slight cutaneous erythema	Very low ⊕ OOO

(*Continued*)

 © 2022. Federação Brasileira de Ginecologia e Obstetrícia. All rights reserved.

Table 1 (*Continued*)

Author (year)	Country	Design	Follow-up	Mean age (years)	N	Inclusion/ exclusion criteria	Interventions	Outcomes measured	Outcomes	Side effects	Certainty[a]
						within the past 5 years, use of HT phytoestrogens, or acupuncture in the last 3 months, endocrine pathologies, psychiatric disease, alcohol or drug addiction, and hypertension/ diabetes mellitus.	acupuncture once a week.			in the area of application of needles.	
**Politano et al.** (2019)[64]	Brazil	RCT	14 weeks	50	72	Inclusion criteria: postmenopausal women over 50 years old. Exclusion criteria: any serious illnesses, malignancy or history of malignancy, and no sign of vaginal atrophy on the assessment examination.	1. CO_2 laser therapy; 2. intravaginal promestriene 10 mg; 3. vaginal lubricant.	Vaginal maturation, VHI score, and FSFI were evaluated at baseline and after 14 weeks of therapy.	CO_2 laser group (mean score 18.68) with promestriene (15.11) and lubricant (10.44) groups ($p < 0.001$). The FSFI score showed improvement in the vaginal elasticity, volume, moisture, and pH in the CO2 laser and promestriene groups.	There were no adverse effects associated with any of the treatments.	Low ⊕⊕ oo

Abbreviations: AEs, adverse events; AI, aromatase inhibitors; ASF, Abbreviated Sexual Function; ASFQ, Abbreviated Sexual Function Questionnaire; BMI, body mass index; CARES, Cancer Rehabilitation Evaluation System; DB, double-blinded; DBCO, double-blinded crossover; DBP, double-blinded placebo-controlled; DBPM, double-blinded placebo-controlled multicenter; DHEA, dehydroepiandrosterone; E_2, estradiol; FDA, Food and Drug Administration; FIEI, Female Intervention Efficacy Index; FSDS-R, Female Sexual Distress Scale-Revised; FSFI, Female Sexual Function Index; FSH, follicle-stimulating hormone; FSQ, Female Sexuality Questionnaire; GAQ, Global Assessment Questionnaire; GSM, genitourinary syndrome of menopause; HRT, hormone replacement therapy. IVE, intravaginal estrogen; IVT, intravaginal testosterone; M, multicenter; MBS, most bothersome symptom; McCoy FSQ, McCoy Female Sexuality Questionnaire; MENQOL, Menopause Specific Quality of life; OLPM, open-labelled placebo-controlled multicenter; PFM, pelvic floor muscles; PGWB, Psychological General Well Being questionnaire; QoL, Quality of Life Questionnaire; RCT, randomized clinical trial; SAQ, Fallowfield Sexual Activity Questionnaire; SERMs, selective estrogen receptor modulators; SFQ, Sexual Function Questionnaire; SIDI-F, Sexual Interest and Desire Inventory–Female; SOC, system organ class; SQoL-F, Sexual Quality of Life-Female; SS, statistically significant; TEAEs, treatment emergent adverse effects; USA, United States of America; VAS, Visual Analog Scale; VHI, Vaginal Health Index; VRS, Verbal Rating Scale; VSI, Vaginal Symptom Index; VVA, vulvovaginal atrophy.
Note: [a]Very low ⊕ ooo; Low ⊕⊕ oo; Middle o⊕⊕⊕ ; High ⊕⊕⊕⊕ . Evaluated using the Grading of Recommendations Assessment, Development and Evaluation (GRADE) classification.[13]

 © 2022. Federação Brasileira de Ginecologia e Obstetrícia. All rights reserved.
